# Molecular analysis of *bla*_SHV_, *bla*_TEM_, and *bla*_CTX-M_ in extended-spectrum β-lactamase producing *Enterobacteriaceae* recovered from fecal specimens of animals

**DOI:** 10.1371/journal.pone.0245126

**Published:** 2021-01-07

**Authors:** Hasan Ejaz, Sonia Younas, Khalid O. A. Abosalif, Kashaf Junaid, Badr Alzahrani, Abdullah Alsrhani, Abualgasim Elgaili Abdalla, Muhammad Ikram Ullah, Muhammad Usman Qamar, Sanaa S. M. Hamam

**Affiliations:** 1 Department of Clinical Laboratory Sciences, College of Applied Medical Sciences, Jouf University, Al Jouf, Saudi Arabia; 2 Department of Pathology, Tehsil Headquarter Hospital Kamoke, District Gujranwala, Pakistan; 3 Faculty of Medical Laboratory Sciences, Omdurman Islamic University, Omdurman, Sudan; 4 Department of Microbiology, Faculty of Life Sciences, Government College University, Faisalabad, Pakistan; 5 Faculty of Medicine, Department of Medical Microbiology and Immunology, Menoufia University, Menoufia, Egypt; 6 Department of Microbiology, King Abdulaziz Specialist Hospital, Sakaka, Saudi Arabia; Nitte University, INDIA

## Abstract

Colonization of extended-spectrum beta-lactamase (ESBL)-producing Enterobacteriaceae as animal gut microbiota is a substantial global threat. This study aimed to determine the molecular characterization of *bla*_SHV_, *bla*_TEM_, and *bla*_CTX-M_ variants in animals, as well as to evaluate the antimicrobial resistance conferred by these genes. We prospectively analyzed 1273 fecal specimens of farm and domestic animals for the isolation of enterobacteria that had the ESBL phenotype by using biochemical methods. The extracted genes were amplified by polymerase chain reaction and sequenced for the characterization of *bla*_SHV_, *bla*_TEM_, and *bla*_CTX-M_ variants. The drug-resistance spectrum and hierarchical clusters were analyzed against 19 antibacterial agents. Out of 245 (19.2%) ESBL enterobacteria, 180 (75.5%) *Escherichia coli* and 34 (13.9%) *Klebsiella pneumoniae* were prevalent species. A total of 73.9% *bla*_CTX-M_, 26.1% *bla*_TEM_, and 14.2% *bla*_SHV_ were found among the enterobacteria; however, their association with farm or domestic animals was not statistically significant. The distribution of *bla* gene variants showed the highest number of *bla*_CTX-M-1_ (133; 54.3%), followed by *bla*_CTX-M-15_ (28; 11.4%), *bla*_TEM-52_ (40; 16.3%), and *bla*_SHV-12_ (22; 9%). In addition, 84.5% of the enterobacteria had the integrons *intI1*. We observed ±100% enterobacteria resistant to cephalosporin, 7 (2.9%) to colistin (minimum inhibitory concentration breakpoint ≥4 μg/mL), 9 (3.7%) to piperacillin-tazobactam, 11 (4.5%) to imipenem, 14 (5.7%) to meropenem, and 18 (7.3%) to cefoperazone-sulbactam, without statistically significant association. Animal gut microbiota contain a considerable number of *bla*_CTX-M_, *bla*_TEM_, *bla*_SHV_, and integrons, which are a potential source of acquired extensive drug resistance in human strains and leaves fewer therapeutic substitutes.

## Introduction

The emergence and transmission of extended-spectrum beta-lactamase (ESBL)-producing *Enterobacteriaceae* from animals has become an important community concern worldwide [1]. Extensive use of antibiotics in human, veterinary, and agricultural treatment has significantly led to the selection and global dissemination of resistant clones in the *Enterobacteriaceae* family over the past years [2]. In particular, animal feed is indiscriminately supplemented with expanded-spectrum antibiotics for prophylaxis and treatment, and sub-therapeutic use of antibacterial drugs in livestock may lead to the dissemination of potentially resistant strains of bacteria in the environment, which poses a serious threat to human health. This practice is illegal in many countries, such as the EU-member states [3]. For example, transmission of ESBL-producers in recent years has become a rampant global threat and is associated with prolonged hospital stays due to severe morbidity [4].

The synthesis of beta-lactamases in gram-negative pathogens remains a significant contributor to beta-lactam resistance, and ESBL-producers pose a substantial threat to many classes of antibiotic, particularly cephalosporins [5]. Clinical pathogens are not the only source of ESBL-producers, as these strains can colonize as gut microbiota in humans and animals. Other environmental sources of transmission of these bacterial strains include food products, water, and sewage [6]. The organisms belong to *Enterobacteriaceae* and produce ESBLs, which hydrolyze cephalosporin and monobactam classes of antibiotics, making them resistant to these antibiotics. These superbugs have been isolated from not only humans but also domestic and farm animals. Pathogenic bacteria from various sources (humans, livestock, industry, and soil) can merge in aquatic habitats, thus increasing the sharing of genes and genetic mechanisms for antibiotic resistance, such as plasmids, transposons, and integrons. This situation may also lead to the transformation of non-pathogenic bacteria into resistant reservoirs in the natural bacterial ecosystem [7]. Various genetic mechanisms are involved in the acquisition and dispersion of antimicrobial resistance (AMR). For example, ESBL genes and other antibiotic-resistance genes can incorporate as a cassette in integrons, which are DNA components that can gather and transfer these cassettes as multidrug-resistant (MDR) mobile genetic elements located on plasmids [8].

*Enterobacteriaceae* acquire ESBL genes by mutation or horizontal transfer of plasmids, which results in oxyimino-cephalosporin resistance. The most common ESBL-encoding genes are *bla*_CTX-M_, *bla*_TEM_, *bla*_SHV_ and *bla*_OXA_ [9]. The occurrence of these genes in enteric bacteria leads to the propensity of these organisms for expanded resistance to various beta-lactam drugs [10]. The ESBL enzymes of the TEM and SHV families are the mutant forms of beta-lactamases, while the CTX-M family originated from environmental bacteria. In addition, several variants of *bla*_CTX-M_ have developed because of point mutations in the gene. ESBL-harboring bacteria can secrete CTX-M, TEM, and SHV enzymes, which collectively have more than 450 variants [11]. Until 2000, SHV and TEM remained the predominant variants of ESBL; however, CTX-M enzymes have taken their place over the last decades [12].

The emergence of ESBL-producing *Escherichia coli* has been reported in cattle meat, fish, and milk in Europe, Asia, and other parts of the world [13]. We aimed in this study to determine the occurrence and molecular characterization of *bla*_SHV_, *bla*_TEM_, and *bla*_CTX-M_ type ESBL variants among the *Enterobacteriaceae* family recovered from the fecal microbiota of companion and farm animals. The study also focused on the spectrum of AMR conferred by the acquisition of these genes. The situation becomes worrisome with the presence of integrons that can transfer a gene cassette of MDR genes, leading to extensively drug-resistant species.

## Materials and methods

### Study design and sampling

**Fig 1:**
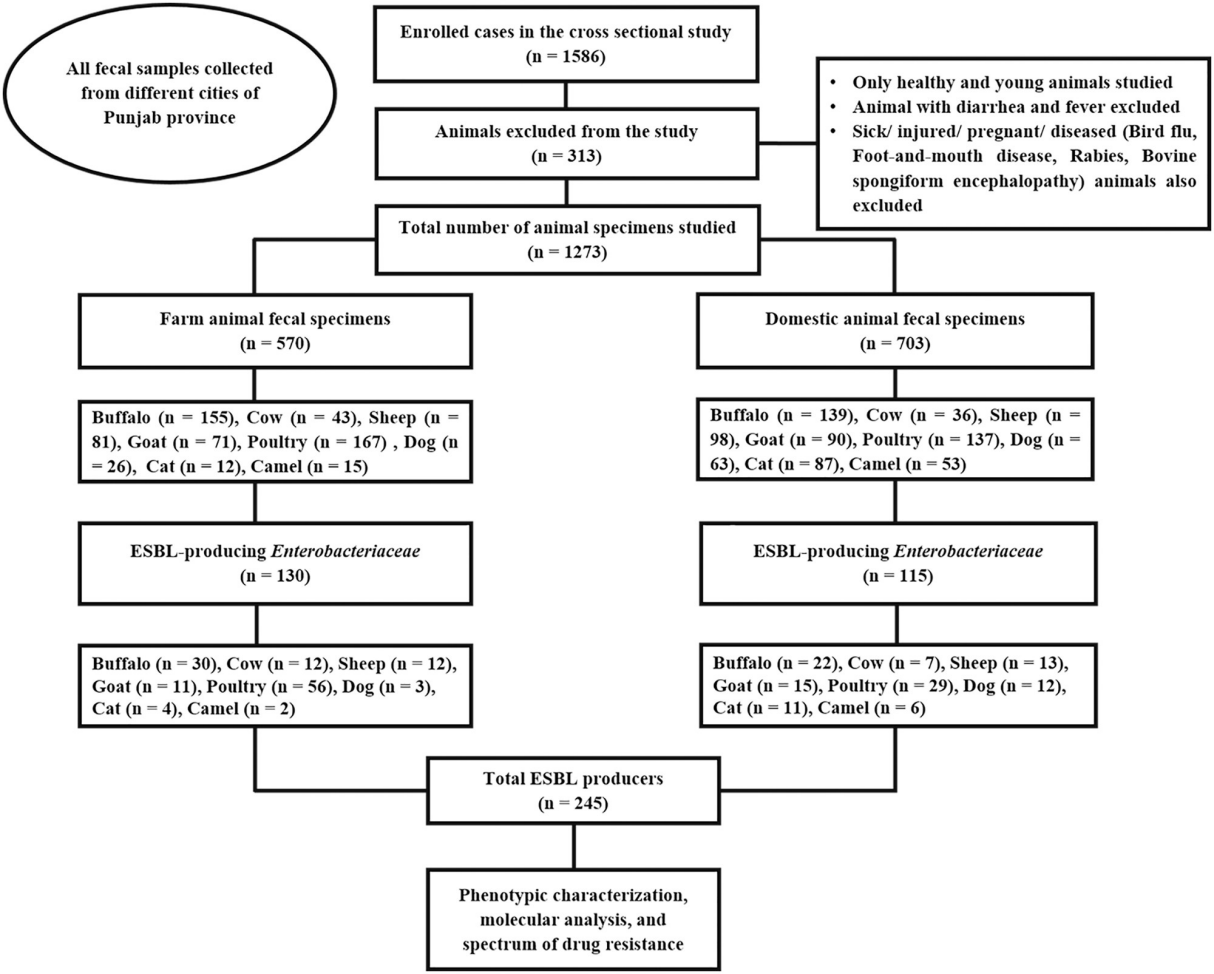
Flow chart of the sampling framework and the processing of fecal specimens.

The fecal specimens without animal intervention prospectively collected after ethical approval following the World Medical Association (WMA) Declaration of Helsinki (Brazil, October 2013) involving human and animal experiments. The ethical committee of the Test Zone Clinical Lab and Diagnostic Centre Lahore, Pakistan, approved the study. All the animals raised in the commercial farms and had contact with other neighboring animals were kept under the category of farm animals. Domestic animals included all animals that were raised and bred in homes. It is a common practice in Pakistani rural areas to raise individual animals at home. A total of 1586 specimens were collected consecutively from the farm and domestic animals in 10 districts of Punjab (Faisalabad, Gujranwala, Gujrat, Hafizabad, Kausar, Lahore, Mandi Bahauddin, Multan, Sahiwal, and Sheikhupura), Pakistan. We further processed 1273 specimens based on the inclusion criteria mentioned in the sampling framework depicted in Fig 1. Approximately 5 g fecal matter per specimen was aseptically collected on a sterile swab and transported in a tube containing Cary Blair transport media for the isolation of *Enterobacteriaceae*.

### Microbiological specimen processing and screening

Each of the fecal swabs was mixed with 10 mL peptone water and incubated overnight at 37°C. A serial dilution of the peptone culture was prepared, and 0.1 mL of suspension from the 10^−5^ dilution was cultured on MacConkey agar (2 mg/L cefotaxime) for the initial selection of ESBL-producing enterobacteria and incubated at 37°C for 18–24 hours. Initially, screened ESBL-producing bacterial growth was processed further for identification [14].

#### Isolation and confirmation of *Enterobacteriaceae*

Enterobacteria were phenotypically characterized by oxidase negativity, colonial morphology, and biochemical strip API 20E (bioMeriéux, France). Bacterial growth other than *Enterobacteriaceae* was excluded from the study.

#### Confirmation and storage of ESBL-producers

**Fig 2:**
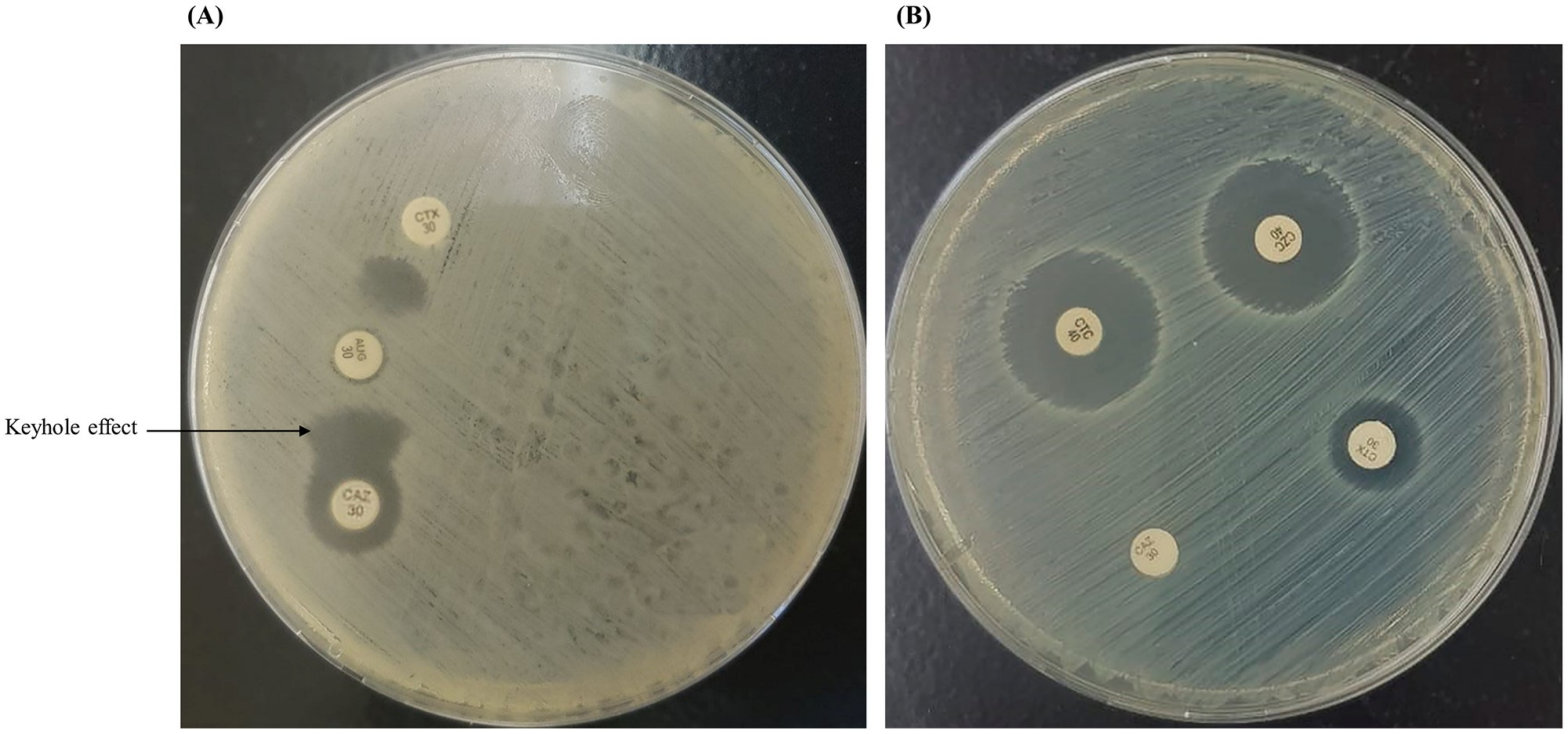
Phenotypic confirmation of ESBL-production. (A) Clavulanate-mediated zone enhancement is observed as a keyhole effect in the double-disk synergy test (DDST) by placing the 30 μg co-amoxiclav (AUG) at a 20 mm distance from the 30 μg ceftazidime (CAZ) and 30 μg cefotaxime (CTX). (B) Combined-disk test (CDT) showing an enhanced zone of ≥5 mm using 40 μg cefotaxime-clavulanate (CTC) and 40 μg ceftazidime-clavulanate (CZC) in comparison with 30 μg cefotaxime (CTX) and 30 μg ceftazidime (CAZ), respectively.

The phenotypic characterization of ESBL-producing strains of *Enterobacteriaceae* was performed on Mueller Hinton by a double-disk synergy test (DDST) and combined-disc test (CDT). Both of these tests use cephalosporin antibiotics individually and in combination with clavulanate [15]. The agar plates were incubated overnight at 35°C-37°C. The beta-lactamase resistant clavulanate mediates a keyhole or a zone of enhancement to visualize the results phenotypically (Fig 2). The bacterial strains of *Enterobacteriaceae* were preserved in brain heart infusion broth supplemented with 15% glycerol and stored at -85°C until used.

#### Determination of antibacterial resistance

The well-isolated colonies of *Enterobacteriaceae* were picked up with a sterile loop and emulsified in normal saline (0.9% sodium chloride) to produce a suspension equivalent to the 0.5 McFarland standard to report the antibacterial drug resistance in vitro. Antibiotic disks belonging to the cephalosporin (30 μg each of the ceftazidime, ceftriaxone, cefotaxime, cefuroxime, cefepime, and 10 μg cefpodoxime), cephamycin (30 μg cefoxitin), aminoglycoside (30 μg amikacin, 10 μg each of the gentamicin and tobramycin), fluoroquinolone (5 μg ciprofloxacin), monobactam (30 μg aztreonam), and carbapenems (10 μg each of imipenem and meropenem) were used. Other antibiotics used in the study included co-trimoxazole (1.25/23.75 μg), co-amoxiclav (20/10 μg), cefoperazone-sulbactam (75/30 μg), and piperacillin-tazobactam (100/10 μg). The suspension was streaked with a sterile swab onto a petri plate containing Mueller Hinton agar and incubated at 37°C for 18 hours after the acclimation of various antibiotic disks. The organisms were reported as sensitive, intermediate sensitive, or resistant according to the zones of inhibition provided by the Clinical and Laboratory Standards Institute (CLSI) [16]. E-test strips (Liofilchem^®^, Italy) were used to determine the minimum inhibitory concentrations (MICs) of colistin with a breakpoint of ≤2 μg/mL for the susceptible strains [16].

#### Quality control bacterial strains

Quality control (QC) strains of American Type Culture Collection (ATCC), *Klebsiella pneumoniae* (700603, ESBL-positive), and *E*. *coli* (25922, ESBL-negative) were used to ensure the quality of bacterial cultures and in vitro antibacterial sensitivity testing following CLSI guidelines [15]. An enhancement zone of ≥5 mm for ceftazidime-clavulanate and ≥3 mm for cefotaxime-clavulanate in comparison with ceftazidime and cefotaxime alone, respectively, was taken as ESBL-positive QC *K*. *pneumoniae* (700603) in CDT. A zone size of ≤2 mm enhancement for the same disks alone and in combination with clavulanate was taken as ESBL-negative QC *E*. *coli* (25922) in CDT [16].

### DNA Extraction and molecular analysis

The DNA templates were extracted from the overnight cultures of ESBL-producing enterobacteria grown on the nutrient agar. The bacterial colonies were mixed in 400 μL TE buffer, and turbidity was optimized with reference to the 0.5 McFarland standard. The DNA from each specimen was extracted by the boiling method from the lysed bacterial strains, and concentration was measured using a NanoDrop spectrophotometer. The supernatant was stored at -20°C and used for the amplification of the genes [17].

#### Genotyping of targeted ESBL genes

The extracted template was primarily amplified to detect *bla*_SHV_, *bla*_TEM_, and *bla*_CTX-M_ using polymerase chain reaction (PCR). The amplification of the genes was performed using our designed primers of *bla*_SHV_ (F: ATTTGTCGCTTCTTTACTCGCC; R: TTCACCACCATCATTACCGACC) and *bla*_TEM_ (F: GTGCGCGGAACCCCTATT; R: GGGATTTTGGTCATGAGATTATC). Primer3 software (http://primer3.wi.mit.edu/) was used to design the primers; the best set of primers based on primer length, melting temperature, and product length was selected and further validated by checking the alignment of designed primers against the targeted nucleotide sequence utilizing the NCBI database BLAST. Prior to synthesizing, these primers were also analyzed for the secondary structures such as hairpins and dimers using Net Primer (http://www.premierbiosoft.com/netprimer/). ATCC strains mentioned in the QC sections were used to establish wet lab QC. CTX-M group variants were amplified using the earlier described specific primers [18,19]. DreamTaq 2× PCR Master Mix (ThermoFisher Scientific, USA) was used to amplify the genes. A 0.5 μM final primer concentration of forward and reverse primers was used in the amplification of the genes, and the reaction was performed using 2 μL of the template and 25 μL Master Mix. The reaction volume was adjusted to 50 μL by the addition of 1.5 μL DMSO and molecular grade distilled water. The cycling protocol optimized with gradient PCR was initiated with denaturation for 2 minutes at 95°C, followed by 33 cycles of 15 seconds at 95°C, 35 seconds at 56°C, and 35 seconds at 72°C, and eventual extension for 7 minutes at 72°C. Additionally, we amplified the Integron I, II (multiplex PCR), and III by using the same reaction volumes with the already reported primers [20]. The reaction was initiated with denaturation for 4 minutes at 95°C, followed by 29 cycles of 45 seconds at 95°C, 50 seconds at 58°C, and 90 seconds at 72°C, and eventual extension for 5 minutes at 72°C.

#### Detection and sequential analysis

Agarose gel was prepared at a concentration of 1.5% mixed with SYBR Safe dye (diluted 10,000×). The amplified gene products were mixed with a 6× loading buffer and loaded on a horizontal gel electrophoresis system at 100 V current for 45 minutes. A 100 bp DNA ruler was used to compare the bands of DNA products with the Gel Documentation System. The PCR products were outsourced for the reverse and forward gene sequencing and analyzed by FinchTV v. 1.4 (Geospiza, Inc.). Other programs, BlastN, BlastP (NCBI), and ExPASy (SIB Group) tools, were used for the nucleotide, amino acid, and translational analysis to report the *bla* gene variants.

### Statistical analysis

GraphPad Prism 6, SPSS v. 23, and BioVinci 1.1.5 were used to perform the data analysis, and p-values <0.05 were taken as significant. Descriptive statistics used to describe the frequencies and percentages of all categorical variables. Odds ratios of ESBL-producing enterobacteria from the farm and domestic animals were calculated using binary logistic regression analysis. The Chi-square test was used to see the relationship of isolates with antimicrobial resistance and the presence of *bla*_SHV_, *bla*_TEM_, and *bla*_CTX-M_ genes.

## Results

### Isolation of enterobacteria and their association with the source

Overall, a noticeable number of ESBL-producers were observed in poultry (85; 34.7%) and buffalo (52; 21.2%), while the remainder of the strains were dispersed in other animals (Fig 1). We detected 245 (19.2%) ESBL-producing strains of enterobacteria from the farm and domestic animals, of which 180 (75.5%) *E*. *coli* and 34 (13.9%) *K*. *pneumoniae* were prominent isolates. The most prevalent microbes from the farm and domestic sources were *E*. *coli* (73% vs. 73.9%), *K*. *pneumoniae* (13.8% vs. 13.9%), and *Enterobacter cloacae* (6.9% vs. 7.8%). The other two less frequent bacteria included *Citrobacter freundii* (3% vs. 2.6%) and *Proteus mirabilis* (3% vs. 1.7%). No significant association was observed when ESBL-producing organisms were compared between farm and domestic sources. The results of the statistical analysis indicated similar odds in both farm and domestic ESBL-producing enterobacteria (Table 1).

**Table 1:** Association of ESBL-producing enterobacteria from the farm and domestic sources (n = 245).

Organisms (n; %)	Farm Source n (%) n = 130	Domestic Source n (%) n = 115	p-value	OR (95% CI)
*E*. *coli* (180; 75.5%)	95 (73)	85 (73.9)	0.9	1.2 (0.59–1.8)
*K*. *pneumoniae* (34; 13.9)	18 (13.8)	16 (13.9)	0.9	1 (0.4–2.07)
*E*. *cloacae* (18; 7.3)	9 (6.9)	9 (7.8)	0.8	1.1 (0.43–2.9)
*C*. *freundii* (7; 2.9)	4 (3)	3 (2.6)	0.8	0.8 (0.18–3.8)
*P*. *mirabilis* (6; 2.4)	4 (3)	2 (1.7)	0.5	0.6 (0.1–3.1)

p-values and odds ratios (OR) were calculated by logistic regression analysis comparing numbers in each row.

### Spectrum of antibacterial drug resistance

**Fig 3:**
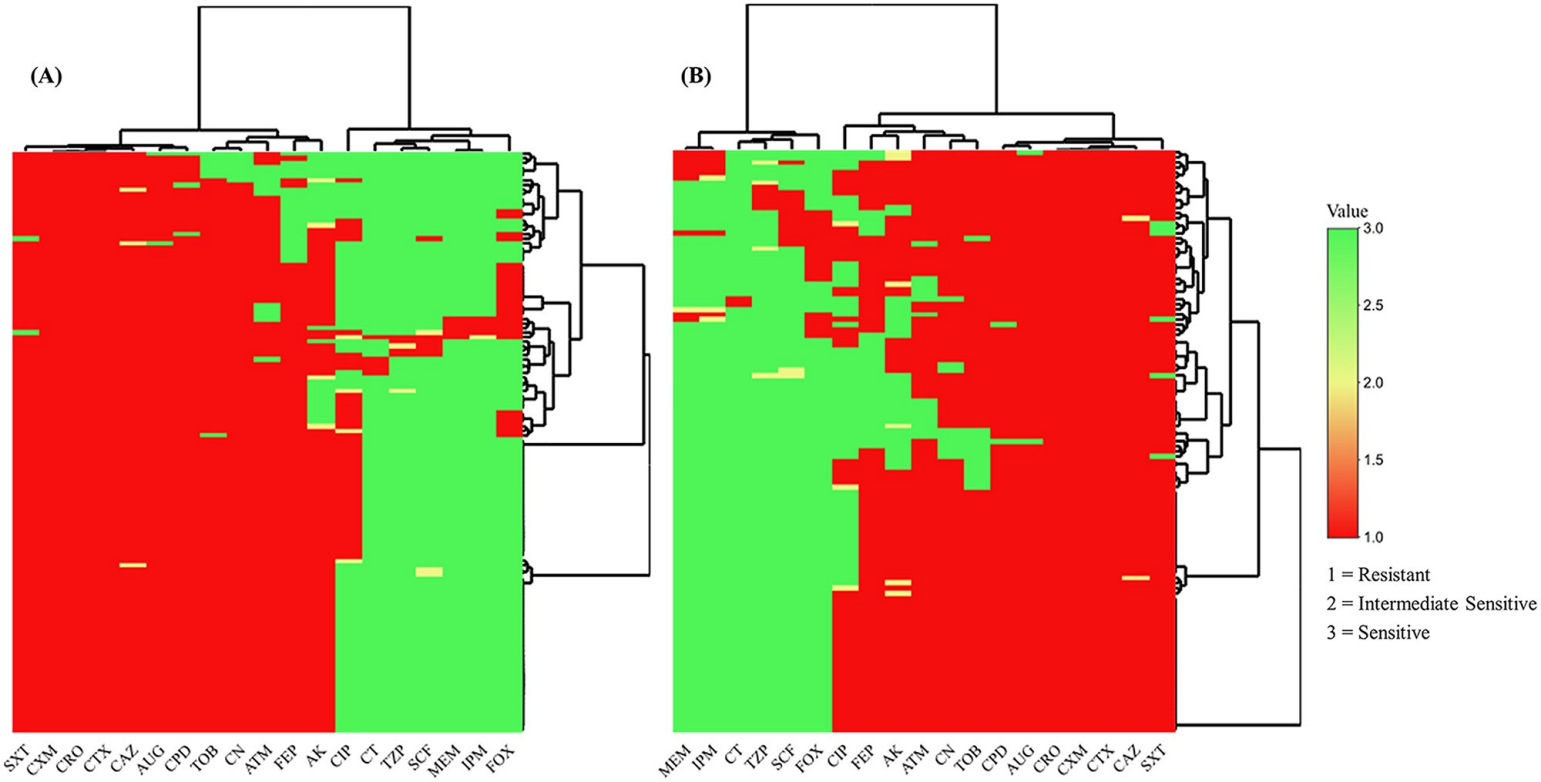
Hierarchical clustering analysis of ESBL-producers. (A) Farm animals. (B) Domestic animals. A total of 19 drugs used for the hierarchical clustering analysis include amikacin (AK), aztreonam (ATM), co-amoxiclav (AUG), ceftazidime (CAZ), ceftriaxone (CRO), cefotaxime (CTX), cefuroxime (CXM), cefpodoxime (CPD), gentamicin (CN), ciprofloxacin (CIP), cefepime (FEP), cefoxitin (FOX), imipenem (IPM), meropenem (MEM), cefoperazone-sulbactam (SCF), co-trimoxazole (SXT), tobramycin (TOB), piperacillin-tazobactam (TZP), and colistin (CT).

The antibacterial drug resistance against the 19 drugs showed that 100% of the bacterial strains were resistant to the various cephalosporin antibiotics. The individual hierarchical clustering of comparison of ESBL-producers isolated from farm and companion animals showed a similar spectrum of drug resistance. Only a few cases isolated from farm animals exhibited pandrug resistance (Fig 3). There were only 5 (2.8%) cases of *E*. *coli* resistant to colistin, 7 (3.9%) to piperacillin-tazobactam, 9 (5%) to imipenem, 12 (6.7%) to meropenem, and 15 (8.3%) to cefoperazone-sulbactam. A similar antibacterial drug resistance profile was observed for *K*. *pneumoniae*, *E*. *cloacae*, *C*. *freundii*, and *P*. *mirabilis*. The overall drug-resistance pattern of enterobacteria revealed minimal resistance (7, 2.9%) to colistin (MIC breakpoint ≥4 μg/mL), 9 (3.7%) to piperacillin-tazobactam, 11 (4.5%) to imipenem, 14 (5.7%) to meropenem, and 18 (7.3%) to cefoperazone-sulbactam. None of the antibiotics showed a statistically significant association with any of the organisms (Table 2).

**Table 2:** Spectrum of drug resistance among the ESBL-producing *Enterobacteriaceae* family.

Drugs Bacterial resistance (n; %)	*E*. *coli* n (%) n = 180	*K*. *pneumoniae* n (%) n = 34	*E*. *cloacae* n (%) n = 18	*C*. *freundii* n (%) n = 7	*P*. *mirabilis* n (%) n = 6	p-value
Amikacin (180; 73.5%)	131 (72.8)	26 (76.5)	13 (72.2)	4 (57.1)	6 (100)	0.6
Aztreonam (218; 89%)	161 (89.4)	29 (85.3)	17 (94.4)	5 (71.4)	6 (100)	0.4
Co-amoxiclav (241; 98.4%)	177 (98.3)	34 (100)	17 (94.4)	7 (100)	6 (100)	0.6
Ceftazidime (240; 98%)	177 (98.3)	32 (94.1)	18 (100)	7 (100)	6 (100)	0.5
Ceftriaxone (245; 100%)	180 (100)	34 (100)	18 (100)	7 (100)	6 (100)	-
Cefotaxime (245; 100%)	180 (100)	34 (100)	18 (100)	7 (100)	6 (100)	-
Cefuroxime (245; 100%)	180 (100)	34 (100)	18 (100)	7 (100)	6 (100)	-
Cefpodoxime (240; 98%)	177 (98.3)	32 (94.1)	18 (100)	7 (100)	6 (100)	0.5
Gentamicin (229; 93.5%)	168 (93.3)	33 (97.1)	17 (94.4)	5 (71.4)	6 (100)	0.2
Ciprofloxacin (99; 40.4%)	74 (41.1)	13 (38.2)	9 (50)	2 (28.5)	1 (16.6)	0.7
Cefepime (193; 78.8%)	142 (78.8)	24 (70.6)	14 (77.7)	7 (100)	6 (100)	0.3
Cefoxitin (46; 18.8%)	32 (17.8)	5 (14.7)	6 (33.33)	3 (42.8)	0 (0)	0.1
Imipenem (11; 4.5%)	9 (5)	0 (0)	2 (11.1)	0 (0)	0 (0)	0.9
Meropenem (14; 5.7%)	12 (6.7)	0 (0)	2 (11.1)	0 (0)	0 (0)	0.8
Cefoperazone-sulbactam (18; 7.3%)	15 (8.3)	2 (5.9)	1 (5.6)	0 (0)	0 (0)	0.9
Co-trimoxazole (237; 96.7%)	173 (96.1)	33 (97.1)	18 (100)	7 (100)	6 (100)	0.9
Tobramycin (225; 91.8%)	165 (91.7)	32 (94.1)	16 (88.9)	6 (85.7)	6 (100)	0.9
Piperacillin-tazobactam (9; 3.7%)	7 (3.9)	1 (2.9)	1 (5.6)	0 (0)	0 (0)	0.9
Colistin (7; 2.9%) ≥ 4 μg/mL	5 (2.8)	1 (2.9)	0 (0)	1 (14.3)	0 (0)	0.4

A Chi-square test was used to calculate p-values.

### Genotypic distribution of *bla*_*SHV*_, *bla*_*TEM*_, and *bla*_*CTX-M*_ genes

**Fig 4:**
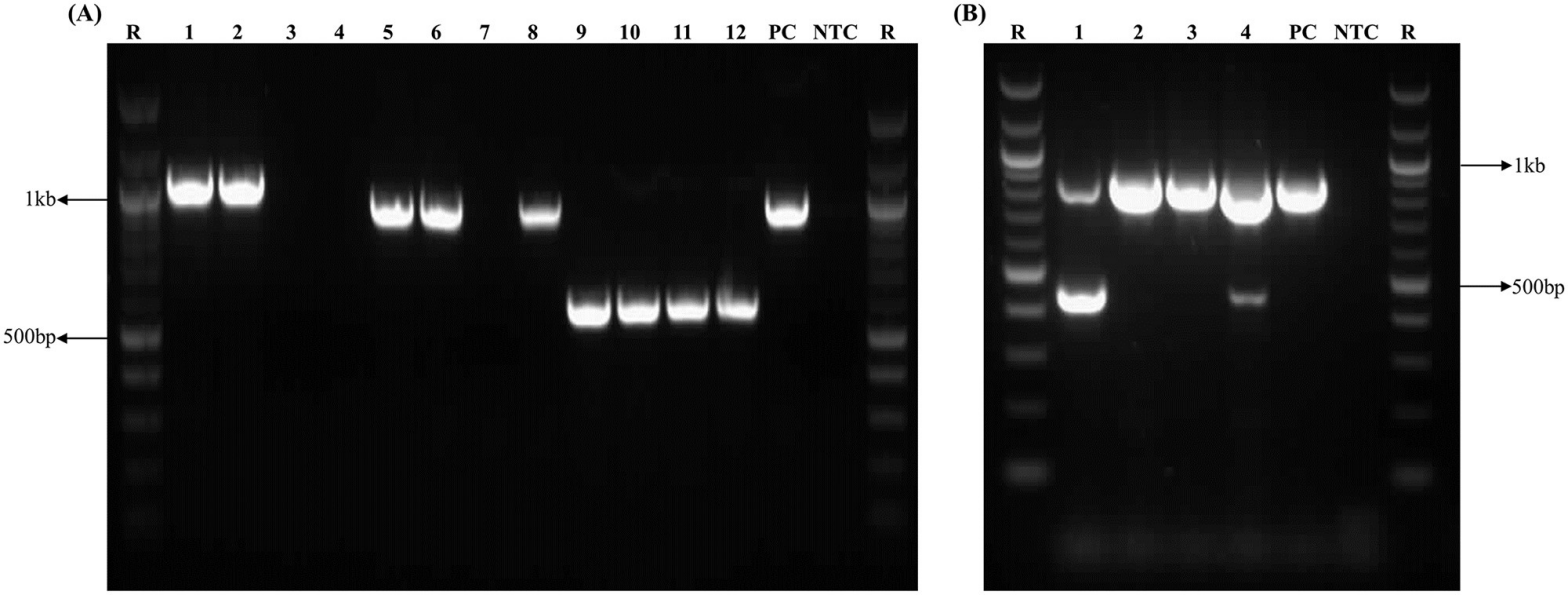
Agarose gel electrophoresis of amplified genes. A 100 bp DNA ruler (R) was used to proximate the gene sizes in agarose gel electrophoresis. Each gel includes a no template control (NTC) and positive control (PC). (A) The amplification of *bla*_TEM_ (1, 2), *bla*_SHV_ (5, 6, 8), and *bla*_CTX-M_ (9–12) genes using the four different DNA templates. No amplified product of *bla*_TEM_ seen at positions 3 and 4, and *bla*_SHV_ at position 7. (B) Multiplex PCR amplification of *intI1* (1, 2, 3, 4) and *intI2* (1, 4) genes.

The distribution of *bla*_SHV_, *bla*_TEM_, and *bla*_CTX-M_ genes varied in ESBL-producing strains of enterobacteria recovered from fecal specimens of companion and farm animals. The co-existence of these genes was found in several bacteria. The extracted DNA amplified by the PCR and the detection of any one of the variants of CTX-M, TEM, and SHV gene products confirmed the presence of ESBL-producing enterobacteria. The most common detected genes in ESBL-producing isolates were *bla*_CTX-M_ (73.9%), followed by *bla*_TEM_ (26.1%) and *bla*_SHV_ (14.2%) (Fig 4A). Of the total 180 *E*. *coli* strains, 134 (74.4%) harbored *bla*_CTX-M_ gene, 47 (26.1%) *bla*_TEM_, and 22 (12.2%) *bla*_SHV_. In *K*. *pneumoniae*, 23 (67.6%) isolates harbored *bla*_CTX-M_, 11 (32.4%) *bla*_TEM_, and 8 (23.5%) *bla*_SHV_. There were 16 (88.9%) *E*. *cloacae* positive for *bla*_CTX-M_, while 3 (16.7%) isolates were positive for each of the *bla*_TEM_ and *bla*_SHV_ genes. The frequency of the *bla*_SHV_, *bla*_TEM_, and *bla*_CTX-M_ genes was similar in *C*. *freundii* and *P*. *mirabilis*. Although a high number of CTX-M genes were detected in *E*. *coli*, their association was not statistically significant in any bacterial isolate (Table 3).

**Table 3:** Genotypic distribution of *bla*_SHV_, *bla*_TEM_, and *bla*_CTX-M_ in ESBL-producers of *Enterobacteriaceae* recovered from fecal specimens of companion and farm animals.

Organisms	CTX-M n = 181 (73.9%)	p-value	TEM n = 64 (26.1%)	p-value	SHV n = 35 (14.2%)	p-value
*E*. *coli* n (%) (n = 180)	134 (74.4)	0.7	47 (26.1)	0.9	22 (12.2)	0.1
*K*. *pneumoniae* n (%) (n = 34)	23 (67.6)	0.4	11 (32.4)	0.4	8 (23.5)	0.1
*E*. *cloacae* n (%) (n = 18)	16 (88.9)	0.1	3 (16.7)	0.3	3 (16.7)	0.7
*C*. *freundii* n (%) (n = 7)	4 (57.1)	0.3	2 (28.6)	0.9	1 (14.3)	1
*P*. *mirabilis* n (%) (n = 6)	4 (66.7)	0.7	1 (16.7)	0.6	1 (16.7)	0.9

p-values indicate the association of each gene with the individual organism and calculated by the Chi-square test.

#### Distribution of *bla* gene variants among the enterobacteria

Overall, the distribution of *bla* gene variants in enterobacteria exhibited the highest numbers for *bla*_CTX-M-1_ (133; 54.3%), *bla*_CTX-M-15_ (28; 11.4%), *bla*_CTX-M-8_ (9; 3.7%), *bla*_TEM-52_ (40; 16.3%), *bla*_TEM-1_ (15; 6.1%), *bla*_SHV-12_ (22; 9%), and *bla*_SHV-28_ (9; 3.7%). A very high distribution of *bla* gene variants in *E*. *coli* showed 101 (56.1%) *bla*_CTX-M-1_, 30 (16.7%) *bla*_TEM-52_, 19 (10.6%) *bla*_CTX-M-15_, and 13 (7.2%) *bla*_SHV-12_. *K*. *pneumoniae* contained 14 (41.2%) isolates with *bla*_CTX-M-1_, 6 (17.6%) *bla*_TEM-52_, and 5 (14.7%) *bla*_CTX-M-15_. A total of 11 (61.1%) *bla*_CTX-M-1_ were isolated in *E*. *cloacae*, followed by 3 (16.7%) each of *bla*_CTX-M-15_ and *bla*_SHV-12_. Overall, the highest number of *bla* variants in enterobacteria belonged to *bla*_CTX-M-1_ (n = 133). The detailed distribution of the *bla* gene variants isolated from all the members of *Enterobacteriaceae* in the study is shown in Table 4.

**Table 4:** Distribution of *bla* gene variants among the various strains of enterobacteria (n = 245).

*bla* gene variants n (%)	*E*. *coli* (n = 180) (73.5%)	*K*. *pneumoniae* (n = 34) (13.9%)	*E*. *cloacae* (n = 18) (7.3%)	*C*. *freundii* (n = 7) (2.9%)	*P*. *mirabilis* (n = 6) 2.4%)
CTX-M-1 (133; 54.3)	101 (56.1)	14 (41.2)	11 (61.1)	4 (57.1)	3 (50)
CTX-M-15 (28; 11.4)	19 (10.6)	5 (14.7)	3 (16.7)	0 (0)	1 (16.7)
CTX-M-8 (9; 3.7)	6 (3.3)	2 (5.9)	1 (5.6)	0 (0)	0 (0)
CTX-M-9 (5; 2)	4 (2.2)	1 (2.9)	0 (0)	0 (0)	0 (0)
CTX-M-2 (3; 1.2)	2 (1.1)	0 (0)	1 (5.6)	0 (0)	0 (0)
CTX-M-14 (3; 1.2)	2 (1.1)	1 (2.9)	0 (0)	0 (0)	0 (0)
TEM-1 (15; 6.1)	10 (5.6)	4 (11.8)	1 (5.6)	0 (0)	0 (0)
TEM-52 (40; 16.3)	30 (16.7)	6 (17.6)	2 (11.1)	1 (14.3)	1 (16.7)
TEM-4 (4; 1.6)	3 (1.7)	1 (2.9)	0 (0)	0 (0)	0 (0)
TEM-10 (3; 1.2)	3 (1.7)	0 (0)	0 (0)	0 (0)	0 (0)
TEM-26 (2; 0.8)	1 (0.6)	0 (0)	0 (0)	1 (14.3)	0 (0)
SHV-12 (22; 9)	13 (7.2)	4 (11.8)	3 (16.7)	1 (14.3)	1 (16.7)
SHV-28 (9; 3.7)	6 (3.3)	3 (8.8)	0 (0)	0 (0)	0 (0)
SHV-11 (4; 1.6)	3 (1.7)	1 (2.9)	0 (0)	0 (0)	0 (0)

### Detection of integrons among the enterobacteria

**Fig 5:**
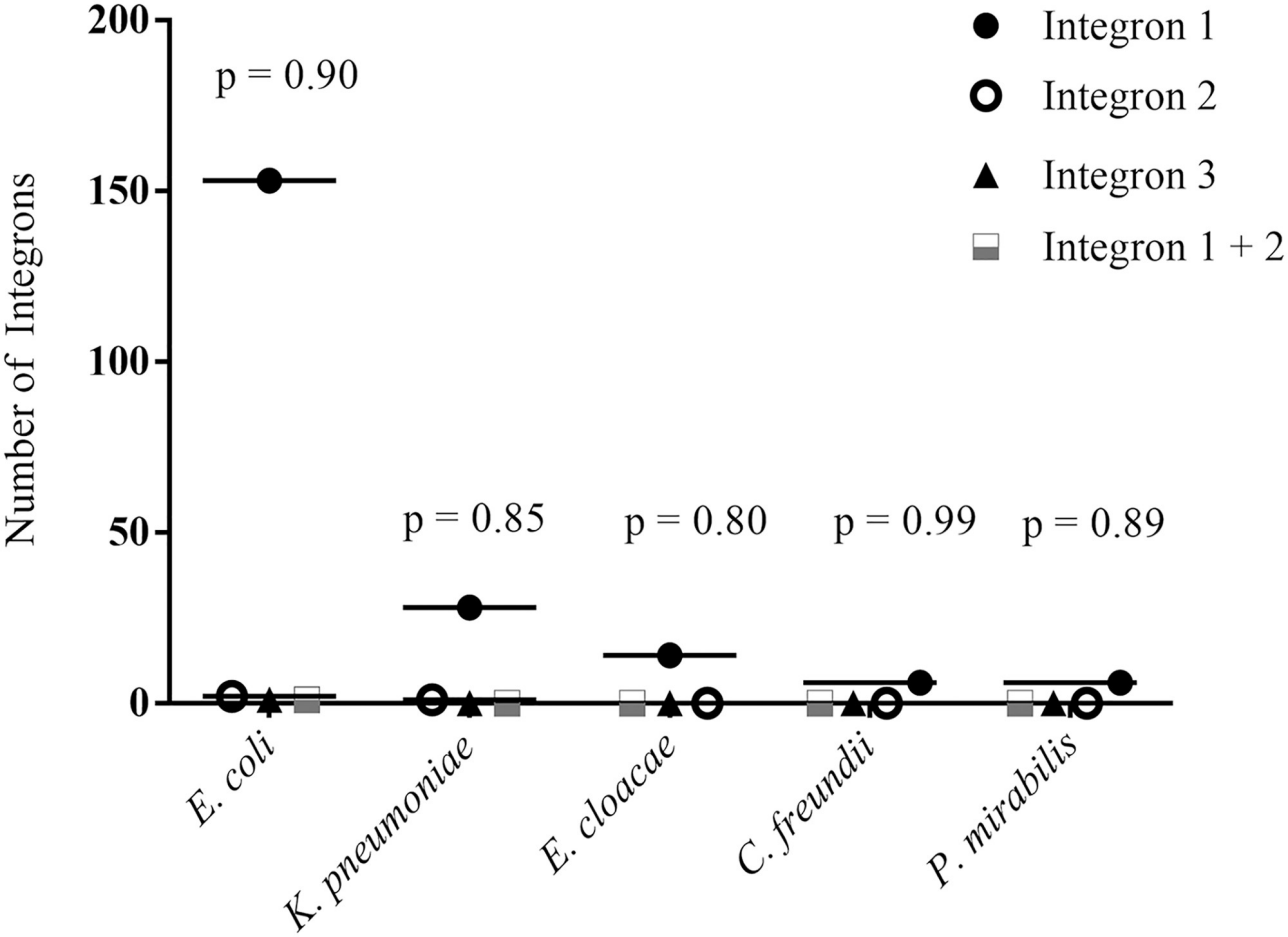
Association of integron genes with ESBL-producing enterobacteria.

A very high number of integrons were observed among the enterobacteria isolated from both the farm and the companion animals (Fig 4B). A total of 207 (84.5%) bacterial strains were found to have *intI1*, and 3 (1.2%) *intI2*. Only 1 (0.4%) case of *intI3* and 1 (0.4%) case of *intI1* + *intI2* (co-existence) were detected in *E*. *coli*. The distribution of class 1 integrons was highest among the *E*. *coli* (153; 62.4%) and *K*. *pneumoniae* (28; 11.4%). The *intI2* was detected only in 2 (0.8%) cases of *E*. *coli* and 1 (0.4%) *K*. *pneumoniae*. A high proportion of class 1 integrons was detected in all isolates, but the difference in the integron distribution was not statistically significant with any of the bacteria (Fig 5).

## Discussion

ESBL-producing enterobacterial strains are a substantial menace for public health due to their expanded cephalosporin and monobactam drug resistance. The presence of these superbugs as normal microbiota in companion and farm animals indicates a potential source of acquired resistance for human pathogens and normal microbiota. Animals are raised as farm and companion (domestic) animals in Pakistan, which are in close contact with the handlers and their families. Currently, there is a paucity of information on ESBL-producing strains from animals and their potential contribution to human antimicrobial resistance. In this study, we investigated these drug-resistance genes and the existence of mobile genetic elements in farm and companion animals. We report the dissemination of ESBL genes in 245 (19.2%) enterobacterial strains, which considerably include 180 (75.5%) *E*. *coli* and 34 (13.9%) *K*. *pneumoniae*. The present study corroborates past reports on ESBL-producing enterobacteria from different regions of the world [21,22]. The prevalence of these strains in our study is relatively higher than in some earlier studies but lower than those previously reported in Nigeria and Algeria [22,23]. ESBL-producing enterobacteria were found to be a little higher (53.1%) among the farm animals than domestic animals (46.9%) in our study. A significant contribution to the dissemination of ESBL-producing strains is the large-scale use of antimicrobials, especially in farms, unskilled workers, and holding healthy and infected animals in close contact. ESBL carriage rate among companion animals could be due to domestic misuse of antimicrobials, close contact with other animals, or the use of contaminated raw food.

Only a few partial reports are available on the disseminations of *bla*_CTX-M_, *bla*_TEM_, and *bla*_SHV_ genes, and integrons, among enterobacteria of healthy domestic and farm animals in Pakistan, and our report is probably the first of its kind. The highest prevalence was observed for *bla*_CTX-M_ (73.9%), followed by *bla*_TEM_ (26.1%) and *bla*_SHV_ (14.2%). The co-existence of these genes was found in several isolates. These findings are somewhat in line with studies from Germany and Nigeria, which reported 69.9% and 44.7% *bla*_CTX-M_ occurrence, respectively [22,24]. However, a Turkish study demonstrated *bla*_TEM_ as the most frequent gene, followed by *bla*_CTX-M_ and *bla*_SHV_ [25]. ESBL-producing strains of *E*. *coli* isolated from healthy animals’ fecal microbiota in Algeria demonstrated 90% *bla*_CTX-M_ [23]. The distribution of *bla*_SHV_, *bla*_TEM_, and *bla*_CTX-M_ genes varies greatly among the enterobacteria of companion and farm animals. In addition to the imprudent antibiotic use, contamination of fecal material in animal feed and inappropriate sanitation may disperse the *bla*_SHV_, *bla*_TEM_, and *bla*_CTX-M_ genes. ESBL genes can be exchanged among the genera of different bacteria, which leads to an uncontrolled dispersion of these genes to many more new bacterial strains [26]. Most of the ESBL genes are detected from *E*. *coli* strains isolated from domestic and farm animals [27]. Our study also highlighted the distribution of *bla* gene variants in enterobacteria and reported that 54.3% of isolates harbored *bla*_CTX-M-1_. Our findings are supported by a German study that reported the highest number of *bla*_CTX-M-1_ (69.9%), *bla*_CTX-M-15_ (13.6%), *bla*_CTX-M-14_ (11.7%), *bla*_TEM-52_ (1.9%), *bla*_SHV-12_ (1.4%), *bla*_CTX-M-3_ (1.0%), and *bla*_CTX-M-2_ (0.5%) [24]. However, the German study was performed in diseased animals compared to healthy animals in our study. Conversely, 80% *bla*_CTX-M-15_, 20% *bla*_SHV-12_, 15% *bla*_CTX-M-1_, and 5% *bla*_TEM-1_ were reported from animal fecal isolates in Algeria [23]. A high prevalence of *bla*_CTX-M-15_ (86%) detected in *bla*_CTX-M_ group, *bla*_SHV-11_ (57%), *bla*_SHV-83_ (29%) and *bla*_SHV-1_ (16%) in *bla*_SHV_ and *bla*_TEM-1B_ (28%) among the *bla*_TEM_ harboring clinical bacterial species in Pakistan [9]. Umair et al. reported 86.2% *bla*_CTX-M-15_ and 6.9% *bla*_CTX-M-1_ type ESBLs in *E*. *coli* recovered from Pakistani humans’ fecal sources, cattle, and poultry. They found two cases of *bla*_CTX-M-55_ from the poultry sources; however, this finding is inconsistent with the observations in our study [28]. The occurrence of ESBL gene variants is present in animal and clinical bacterial isolates in several parts of the world. Cross-country differences in the distribution of these variants have also been reported and discussed in publications from Spain, Nigeria, and Turkey [8,22,25].

A high (34.7%) prevalence of ESBL-producers was found in poultry in our study, and these results corroborate similar studies of chicken production facilities [29,30]. Poultry is one of the most widely consumed products worldwide, and many antibiotics are used during poultry production in many countries. The risk of developing or increasing microbial resistance in the poultry environment may endanger human lives [30,31]. Trends in a study showed that antimicrobials are widely overused in broiler development in Pakistan and urgently need action [32]. Overall, second-highest ESBL-production (29%) was observed in cattle (buffalo and cow), primarily from the farm cattle (17.1%). Antibiotics are conveniently accessible in Pakistan and given to both farm and domestic cattle as growth promoters rather than treatment. The antibiotics have also been used injudiciously in animal growth promotions instead of therapeutic purposes in America [33]. ESBL production among domestic dogs (10.4%) and cats (9.6%) was higher than the animals raised in farms. ESBL-producing strains can be transmitted among humans and other domestic animals, possibly through close human-animal contact. Raw-food given to the domestic animals could serve as a potential source of resistant bacterial strains [34].

A very intriguing finding in our study was the detection of *intI1* in 84.5% of the ESBL-producers, while few cases had *intI2* and *intI3*. The class 1 integrons were ubiquitously reported from enterobacteria from India and China [29,35]. The occurrence of 92% integrons was reported in ESBL-producing *E*. *coli* in Spain, with a significantly higher number of class 1 integrons, followed by class 2 and 3 integrons, which suggested horizontal dissemination of multidrug resistance in bacterial strains [8]. Integrons carry a drug-resistance gene cassette against multiple classes of drugs, and the detection of these mobile gene cassettes in farm and domestic animal fecal microbiota is a cornerstone of multidrug resistance other than cephalosporins.

We detected a broad spectrum of drug resistance to cephalosporins, co-amoxiclav, co-trimoxazole, and aminoglycosides, with substantially lower resistance against colistin, piperacillin-tazobactam, cefoperazone-sulbactam, and carbapenems. Impulsive use of antibiotics in animal feed has led to wider resistance in ESBL-producing enterobacteria to no-cephalosporin antibiotics. Resistance to non-cephalosporin drugs has been reported in Indian Punjab against nalidixic acid, ciprofloxacin, tetracycline, ampicillin, co-trimoxazole, chloramphenicol, and gentamicin [3]. A Pakistani study on various animal and human ESBL-producing *E*. *coli* reported extensive drug resistance to beta-lactam and non-beta-lactam antibiotics. *E*. *coli* species showed minimum resistance to a single antibacterial drug, polymyxin B [28]. We also observed a good in vitro activity of colistin (polymyxin E), which is structurally identical to polymyxin B. A similar antibiogram pattern from Tunisia reported imipenem as the drug of choice against these superbugs [36]. In contrast, broader drug resistance was observed against cephalosporins and other antibiotics in a Korean study carried out on healthy companion animals and cohabitating humans, and ESBL-producing isolates exhibited extremely low or no resistance to carbapenems, colistin, and amikacin [37]. In addition, *E*. *coli* isolates harboring *bla*_CTX-M_, *bla*_TEM_, and *bla*_SHV_ expressed variable resistance to cephalosporins, trimethoprim, and sulfamethoxazole. Nevertheless, none of these isolates displayed resistance to carbapenems, piperacillin-tazobactam, cefoperazone-sulbactam, amikacin, and only 5% of isolates were resistant to chloramphenicol [23]. Contrary to these results, ESBL-producing strains in our study showed 73.5% antimicrobial resistance to amikacin.

The presence of ESBL genes makes the bacteria resistant to several useful empirical regimens and leaves few therapeutic strategies [38,39]. There is no limitation for using antimicrobials in animal food, which leads to expanded drug resistance and is a significant challenge in Pakistan [32]. The hierarchical clustering of farm and domestic animals demonstrated extensive antibacterial resistance against several classes of drugs that are not inhibited by ESBLs. The detection of integrons in most of the bacterial isolates advocates the phenomenon of extensive resistance due to these mobile genetic elements.

### Conclusions

Farm and companion animals predominantly contain *bla*_CTX-M_ clusters, which could be animal genesis, causing hasty changes in drug-resistance epidemiology. CTX-M-1 and CTX-M-15 are also commonly found among human pathogens suggesting a common clonal lineage and a potential ESBL dissemination source in farm and domestic animals. These superbugs respond to fewer antibacterials, including colistin, piperacillin-tazobactam, carbapenems, and cefoperazone-sulbactam, which is alarming. The dissemination of integrons among the animal microbiota may diminish the remaining antibiotic options and lead us to the post-antibiotic era, which directs a future epidemiological research perspective.

## Supporting information

S1 Raw images (PDF)
