# Supplementary figures and images for: Molecular analysis of blaSHV, blaTEM, and blaCTX-M in extended-spectrum β-lactamase producing Enterobacteriaceae recovered from fecal specimens of animals
Source: PLoS One. 2021 Jan 7;16(1):e0245126. doi: 10.1371/journal.pone.0245126 (PMC7790543; doi:10.1371/journal.pone.0245126)

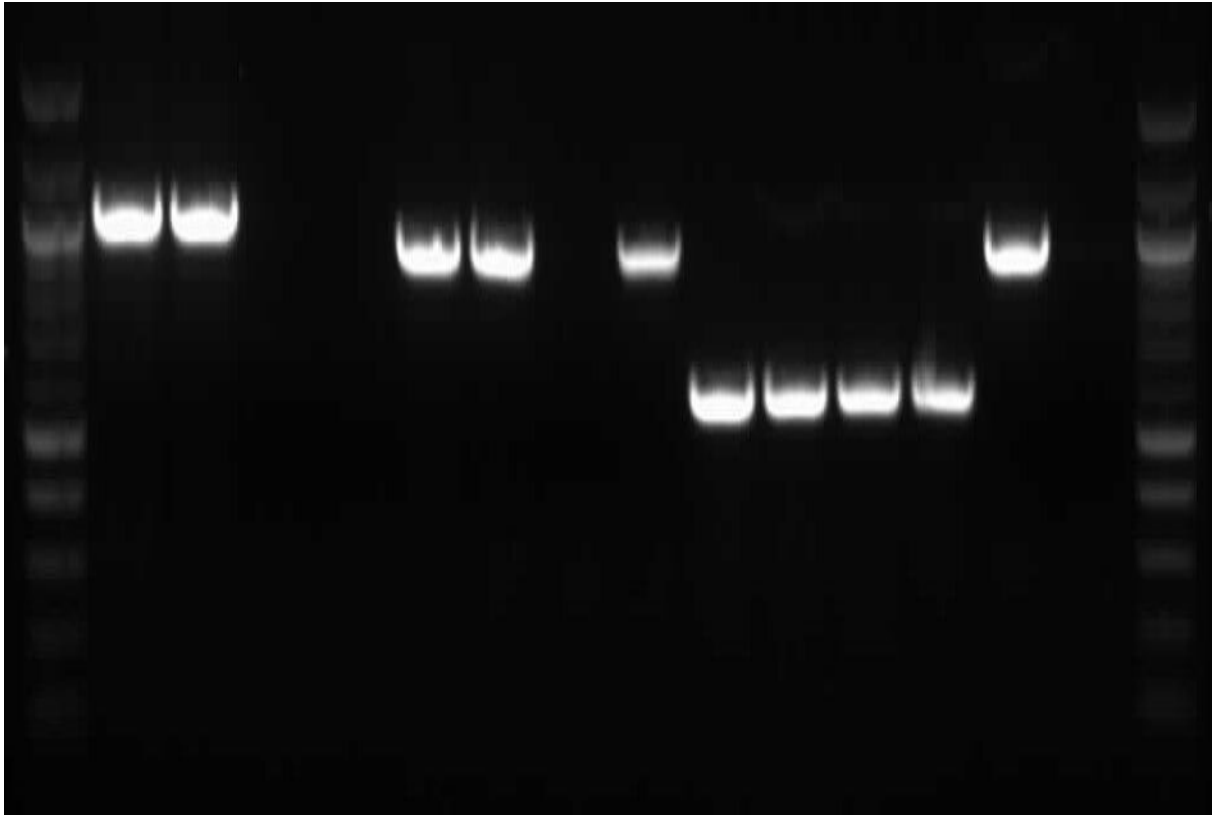

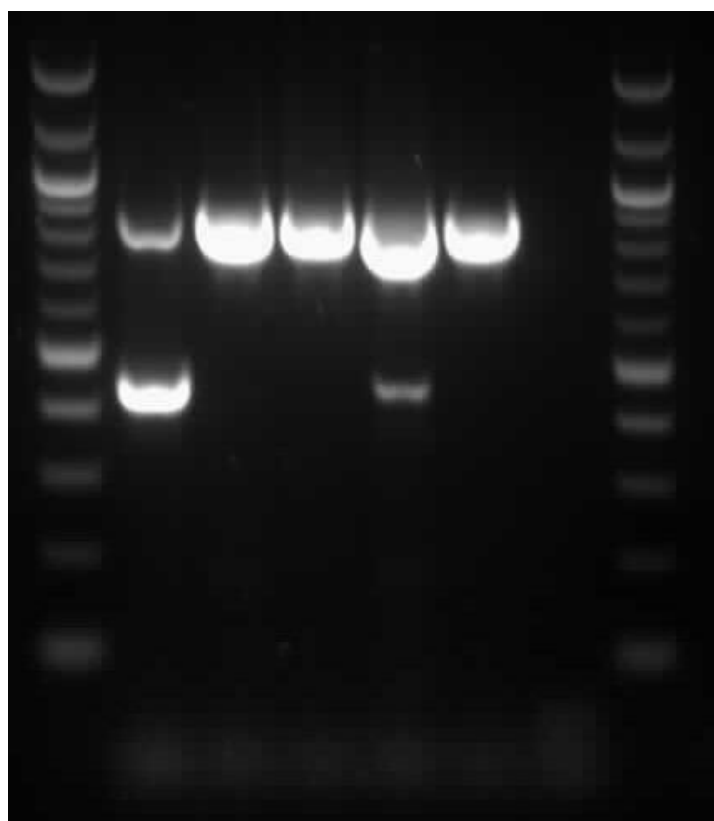

Supplement: S1 Raw images — (PDF) [file pone.0245126.s001.pdf]
